# MISSED INSIGHTS FOR EARLIER MANAGEMENT OF PARKINSON’S DISEASE AND THE VALUE OF DOPAMINE TRANSPORTER (DAT) SCANS

**DOI:** 10.1093/geroni/igad104.3474

**Published:** 2023-12-21

**Authors:** Mohib Hafeez, Amanda Pangle, Jeanne Wei, Gohar Azhar

**Affiliations:** University of Arkansas for Medical Sciences, Little Rock, Arkansas, United States; University of Arkansas for Medical Sciences, Little Rock, Arkansas, United States; University of Arkansas for Medical Sciences, Little Rock, Arkansas, United States; University of Arkansas for Medical Sciences, Little Rock, Arkansas, United States

## Abstract

This retrospective study focused on the role of Dopamine Transporter (DAT) scans in diagnosing Parkinson’s disease (PD) in older adults with cognitive impairment (CI). We reviewed brain imaging of 6,483 individuals, aged 60 and above with CI. 332 had a DAT scan of which 203 were positive and 80/203 (39%) were subsequently started on treatment with carbidopa-levodopa. On the other hand, 243 patients showed Parkinson’s (PD) associated structural changes on CT or MRI including mineral deposition, hypodensities, and lacunar infarcts in dopamine-rich regions such as the caudate, putamen, and basal ganglia but none of these patients had a DAT scan, or treatment with carbidopa-levodopa therapy. 71/243 (29%) of these patients had also experienced falls or had gait disorders. This points towards a potential missed diagnosis of PD, which can respond to therapy in the early stages. Our results suggest that providers may overlook subtle signs of Parkinsonism in patients with CI. This could result in symptom worsening and treatment delay. Since CI is often first brought to the attention of PCPs, our findings call for an increased effort to inform PCPs on the role of DAT scans to aid in the diagnosis of dopamine deficiency states. By having a better understanding of PD-related structural changes seen on brain imaging and then using a DAT scan to confirm dopamine deficiency, treatment for PD might be started earlier or timely referral made to a specialist for comprehensive care, reduction of disability, and improvement of the quality of life of patients.

